# Orthorexia nervosa in gay men—the result of a spanish-polish eating disorders study

**DOI:** 10.1186/s12889-022-14943-7

**Published:** 2023-01-09

**Authors:** Piotr Karniej, Jesús Pérez, Raúl Juárez-Vela, Iván Santolalla-Arnedo, Vicente Gea-Caballero, Pablo del Pozo-Herce, Anthony Dissen, Michał Czapla

**Affiliations:** 1 Faculty of Health Sciences, Group of Research in Care (GRUPAC), University of La Rioja, 26004 Logroño, Spain; 2 Faculty of Finance and Management, WSB University in Wrocław, 53-609 Wrocław, Poland; 3 Institute of Biomedical Research of Salamanca (IBSAL), Psychiatry Unit, Department of Medicine, Universidad de Salamanca, Salamanca, Spain; 4 Department of Psychiatry, University of Cambridge, Cambridge, UK; 5 Cambridgeshire and Peterborough NHS Foundation Trust, Cambridge, UK; 6 Norwich Medical School, University of East Anglia, Norwich, UK; 7 Group of Research in Sustainability of the Health System, Biomedical Research Center of La Rioja (CIBIR), La Rioja, Spain; 8 Faculty of Health Sciences, Valencian International University, Pintor Sorolla 21 46002, Valencia, Spain; 9 Research Group in Community Health and Care (SALCOM), Valencian International University, Valencia, Spain; 10 Department of Psychiatry, Fundación Jiménez Díaz University Hospital, Madrid, Spain; 11 Health Research Institute, Fundación Jiménez Diaz, Madrid, Spain; 12 School of Nursing, Fundación Jiménez Diaz. Autonomous University, Madrid, Spain; 13 Health Science Faculty, Stockton University, Stockton, New Jersey USA; 14 Department of Emergency Medical Service, Wroclaw Medical University, 51-618 Wroclaw, Poland; 15 Institute of Heart Disease, University Hospital, Wroclaw, Poland

**Keywords:** Orthorexia nervosa, Eating disorders, Gay, Stigma, PrEP

## Abstract

**Background:**

The purpose of this exploratory study was to identifying demographic factors and unique predictors of ON e.g., the use of pre-exposure prophylaxis (PrEP), the use of social media and the Grindr ® dating application among a sample group of Spanish and Polish identifying gay men.

**Methods:**

The study was conducted in Poland and Spain between March and June 2021 using questionary: ORTO-15. Data was collected using a three-section self-administered questionnaire. The first section contained demographic data, the second part was the Polish and Spanish version of the Orto-15, and the third part was the Polish and Spanish version of the EAT-26.

**Results:**

Total enrollment was 394 gay men. In regression proportional hazards single model, significant predictors of ON were: age (OR = 0.964, 95% CI, 0.944–0.984), BMI (OR = 0.895, 95% CI, 0.848–0.944), staying in an informal relationship compared to being single (OR = 2.138, 95%CI, 1.225–3.732), occasional use of Pre-exposure Prophylaxis (OR = 4.667, 95%CI, 1.186–18.362) and use of the Grindr application (OR = 5.312, 95%CI, 3.373–8.365). Instagram users had lower risk of ON (OR = 0.479, 95%CI, 0.279–0.822). The multivariate analysis showed that Grindr usages (OR = 4.72; 95%CI, 2.89–7.72) correlated with higher risk of ON. Higher BMI (OR = 0.913, 95%CI, 0.861–0.98) and daily use of Pre-exposure Prophylaxis (OR = 0.142, 95%CI, 0.03–0.674) is associated with lower risk of ON.

**Conclusions:**

The most important predictors of orthorexia nervosa in gay men are: low BMI and the use of Grindr. The effect of daily usage of PrEP is associated with lower risk, and occasional use is associated with increased risk, of orthorexia nervosa.

**Supplementary Information:**

The online version contains supplementary material available at 10.1186/s12889-022-14943-7.

## Introduction

There has been much discussion in recent years about the importance of practicing healthy eating habits for the purposes of reducing the risk of chronic non-communicable diseases (NCD's) like cardiovascular disease, diabetes, and cancer [1]. While the pursuit of healthier ways of eating can be appropriate, clinical practice indicates that behind an apparently healthy dietary pattern there may be other factors at play [2]. One such concern within the field of eating disorders research and practice is Orthorexia Nervosa (ON), which mainly consists of a pathological obsession with eating foods and food products that are considered healthy that leads to following a restrictive diet and a harmful focus on the quality of these products and their origin [2]. While not an officially recognized ED diagnosis within fifth edition of the Diagnostic and Statistical Manual of Mental Disorders (DSM-5), the term orthorexia nervosa was first coined in 1998 as a means of specifically identifying obsessive and ritualistic eating behaviours related to the concept of eating healthfully [3]. Within the academic and clinical discussions surrounding ON, concern stems from what may seem like actions and habits being undertaken to improve one’s dietary habits and achieve best possible health condition, can instead lead a person with ON to serious health complications such as malnutrition, loss or disturbance of relationships with loved ones, and deterioration of quality of life [4].

It is a well-known phenomenon that people who consider themselves as part of the LGBTQ community (hereinafter also referred to as: LGBT) (lesbian, gay, bisexual, transgender, queer) experience disproportionate burdens of physical and mental health issues when compared to the heterosexual population [5, 6]. One additional area of disproportionate burden may be in the rates of ED experiences by LGBT individuals, who have been shown to often experience higher rates of eating disorders than their heterosexual counterparts [7]. Factors such as past sexual trauma and abuse [7], body dissatisfaction [8], and the pursuit of an idealized and sexually attractive physical appearance [9] appear to be particularly pronounced among LGBT individuals. These may also increase one’s risk for ON, as factors such as poor body image and a drive for a particular body size have been associated with increased risk for ON [10].

A review of the current literature shows little scholarly work pertaining to the prevalence of ON in gay men, which further contributes to the potential increase in health disparities experienced by gay identified men due to an absence of scholarly inquiry. Without a proper understanding of the potential rates of ON in gay men, health professionals who work with gay men may not be able to assess for and address ON-related behaviours and risk factors appropriately. It should also be added that LGBT patients tend to remain silent about important health problems more often, as they are afraid that disclosing them may lead to stigmatization or even hatred [11]. This increased level of stigma experience, or fear of potential stigma, may further exacerbate this issue. Given the gap in the current literature pertaining to ON in gay men, as opposed to other forms of ED, it is important to understand not only the rates of ON in the gay male population, but also how unique lifestyle factors and personal demographic factors may be playing a contributing role.

Therefore, the aim of this exploratory study was: 1) to identifying demographic factors associated with ON behaviour (measured with the ORTO-15 test) among a sample group of Spanish and Polish identifying gay men; 2) to identifying unique predictors of orthorexia nervosa in gay men.

## Material and methods

### Sample

This study was conducted in Poland and Spain between March and June 2021. After being fully informed of the purposes of the study and providing informed consent, participants were asked to complete an anonymous questionnaire. Inclusion criteria were the ability to read and write in Polish/Spanish, an age of 18 years or older, a gender identity as male, and a sexual orientation identity as gay/homosexual. The total final enrollment was 394 gay men (188 from Poland and 206 from Spain).

### Procedure

The call for this study was distributed via social media advertisements posted in groups for gay men on Facebook and other social media (Instagram, Twitter). In both Poland and Spain the survey was available via an online platform webankieta.pl [12] to obtain data and access control of the sample using IP filtering. The survey was started 436 times and completed by 394 participants (90%).

The data were collected using a three-section self-administered questionnaire. The first section contained questions pertaining to demographic data, such as age, sex, height, body weight, place of living, employment status, relationship status, educational level, eating pattern used, self-reported health assessment, use of Pre-Exposure Prophylaxis (PrEP), and use of social media and dating app (Grindr). The second part was the Polish and Spanish version of the ORTO-15 (Orthorexia Nervosa: ORTO-15). The third part was the Polish and Spanish version of the EAT-26 (Eating Attitudes Test EAT-26).

### Eating disorders tools

The ORTO-15 questionnaire is a self-report 15-item measure with a 4-point Likert scale (always-often-sometimes-never) developed in 2004 by Donini and colleagues, modeled on the Bratman test. Is the most frequently used tool to assess severity of ON [13]. In our study we used the Polish and Spanish validation versions of ORTO-15 [14, 15]. This questionnaire measures the interrelationship between cognitive-rational, clinical and emotional aspects of eating behavior [15]. The ORTO-15 questionnaire assesses beliefs about attitudes covering food selection, the extent to which food concerns influence daily life, the perceived effects of eating healthy food, and habits of food consumption. Lower overall scores refer to more ON components (increased ON tendency). Cronbach's alpha is 0.80 (Spanish version) and 0.78 (Polish version) [13, 14]. Two cut-offs for ORTO-15 have been proposed: < 40 (sensitivity 100%, specificity 73.6%, positive predictive value 17.6%, negative predictive value 100%) and < 35 (sensitivity 86.5%, specificity 94.2%, negative predictive value 94.1%) [14]. Other studies used median split to define individuals with or without ON tendencies [16]. Since our study involved samples recruited in Poland and Spain, the cutoff of 35 was used to identify possible ON, as described in the literature. [16–18]^ (p26)^

The Eating Attitude 26-item questionnaire (EAT-26) is divided into three subscales: Bulimia Nervosa, dieting behaviour, and food preoccupation [19]. The questionnaire consists of 26 items, rated on a 6-point Likert scale (3 = Always, 2 = Usually, 1 = Often, 0 = Sometimes, 0 = Rarely, 0 = Never). Scores above 20 points indicate a real risk of EDs. Higher results obtained by the addition of all 26 scores indicate higher risks of developing eating disorders. In our sample we use a Polish and Spanish validated tools [20, 21]. This test is not a diagnostic tool, but some authors suggested that the EAT-26 might identify cases at risk for EDs in clinical spectrum [20].

### Ethical considerations

This study was approved by the independent Bioethics Committee of the Wroclaw Medical University (decision no. 812/2020) and Committee on Ethics and Research Medicinal of La Rioja (decision no. CEImLAR P.I. 471). The study was carried out in accordance with the tenets of the Declaration of Helsinki and recommendations of good clinical practice. For reporting, the Strengthening the Reporting of Observational Studies in Epidemiology (STROBE) guidelines were followed [22]. All participants gave their informed consent to participate in this study.

### Statistical analysis

The mean, median, standard deviation and quartiles were calculated as a summary of the distribution of quantitative variables, while qualitative variables were summarised with absolute value and percentage. Logistic regression was used to analyse the impact of quantitative variables on the dichotomous outcome. Based on the simple regressions, variables were selected for inclusion in the multiple regression. Variables with the lowest p-values were chosen so that the EPV (Events Per Variable) index was at least 10 [23, 24]. A total of 13 variables were included in the final analysis. Odds ratios (OR) with 95% confidence intervals are shown. The two-sided *P* value < 0.05 was considered as significant, and statistical analysis was performed with R 4.1.2.

## Results

Total final enrollment was 394 gay men. The Polish sample was comprised of 188 adults gay men with a median age of 32 years old (SD 8,58; range 18–62). The Spanish sample was comprised of 206 adults gay men with a median age of 43 years old (SD 10,56; range 18–67). The sample mean age was 38.08 years and mean body max index (BMI) was 26.11 kg/m2. The full group profile with a comparison of the analysed characteristics of the Polish and Spanish Sample is shown in Table 1.

**Table 1:** Characteristics of the group with a comparison of polish and Spanish sample

Parameter		Nationality			p
		**Polish (*N* = 188)**	**Spanish (*N* = 206)**	**Total (*N* = 394)**	
Age [years]	mean ± SD	32.54 ± 8.58	43.14 ± 10.56	38.08 ± 11.01	*p* < 0.001 ^*^
	median	31	43	37	
	quartiles	26.75—37	34—51	29—45	
BMI [kg/m^2^]	mean ± SD	24.56 ± 3.75	27.53 ± 5.04	26.11 ± 4.71	*p* < 0.001 ^*^
	median	24	26.2	24.9	
	quartiles	22.17—26.63	23.92—30.43	22.92—28.37	
Residence	Village	12 (6.38%)	8 (3.88%)	20 (5.08%)	*p* = 0.025 ^*^
	City up to 20 th. inhab	4 (2.13%)	2 (0.97%)	6 (1.52%)	
	City 20–100 th. inhab	18 (9.57%)	10 (4.85%)	28 (7.11%)	
	City 100–500 th. inhab	38 (20.21%)	28 (13.59%)	66 (16.75%)	
	City over 500 th. inhab	116 (61.70%)	158 (76.70%)	274 (69.54%)	
Education	Primary	9 (4.79%)	6 (2.91%)	15 (3.81%)	p < 0.001 ^*^
	Secondary	44 (23.40%)	6 (2.91%)	50 (12.69%)	
	Bachelor's degree	27 (14.36%)	52 (25.24%)	79 (20.05%)	
	Master	93 (49.47%)	138 (66.99%)	231 (58.63%)	
	Doctorate	15 (7.98%)	4 (1.94%)	19 (4.82%)	
Employment status	Full-time paid job	115 (61.17%)	188 (91.26%)	303 (76.90%)	*p* < 0.001 ^*^
	Part-time paid job	6 (3.19%)	4 (1.94%)	10 (2.54%)	
	Own business	33 (17.55%)	6 (2.91%)	39 (9.90%)	
	Pension	2 (1.06%)	2 (0.97%)	4 (1.02%)	
	Unemployed	6 (3.19%)	4 (1.94%)	10 (2.54%)	
	Student	26 (13.83%)	2 (0.97%)	28 (7.11%)	
Marital status	Single	122 (64.89%)	170 (82.52%)	292 (74.11%)	*p* < 0.001 ^*^
	In a partnership	56 (29.79%)	6 (2.91%)	62 (15.74%)	
	Divorced	3 (1.60%)	8 (3.88%)	11 (2.79%)	
	Married to a man	5 (2.66%)	20 (9.71%)	25 (6.35%)	
	Married to a woman	2 (1.06%)	2 (0.97%)	4 (1.02%)	
Nutrition model	Omnivore	158 (84.04%)	202 (98.06%)	360 (91.37%)	*p* < 0.001 ^*^
	Vegan	2 (1.06%)	2 (0.97%)	4 (1.02%)	
	Vegetarian	18 (9.57%)	0 (0.00%)	18 (4.57%)	
	Other	10 (5.32%)	2 (0.97%)	12 (3.05%)	
Self-reported Health assessment	I am healthy, I do not use healthcare	135 (71.81%)	182 (88.35%)	317 (80.46%)	*p* < 0.001 ^*^
	I am ill with chronic diseases, I use healthcare	26 (13.83%)	18 (8.74%)	44 (11.17%)	
	I have health problems but I do not receive health care	12 (6.38%)	4 (1.94%)	16 (4.06%)	
	I have mental health problems, I use psychological or psychiatric help	15 (7.98%)	2 (0.97%)	17 (4.31%)	
PrEP use	Never	169 (89.89%)	197 (95.63%)	366 (92.89%)	*p* = 0.08
	Always, once a day	12 (6.38%)	6 (2.91%)	18 (4.57%)	
	Occasionally	7 (3.72%)	3 (1.46%)	10 (2.54%)	
Facebook use	No	29 (15.43%)	66 (32.04%)	95 (24.11%)	*p* < 0.001 ^*^
	Yes	159 (84.57%)	140 (67.96%)	299 (75.89%)	
Instagram use	No	35 (18.62%)	30 (14.56%)	65 (16.50%)	*p* = 0.344
	Yes	153 (81.38%)	176 (85.44%)	329 (83.50%)	
Twitter use	No	148 (78.72%)	182 (88.35%)	330 (83.76%)	*p* = 0.014 ^*^
	Yes	40 (21.28%)	24 (11.65%)	64 (16.24%)	
Grindr use	No	96 (51.06%)	134 (65.05%)	230 (58.38%)	*p* = 0.007 ^*^
	Yes	92 (48.94%)	72 (34.95%)	164 (41.62%)	
Other social media use	No	173 (92.02%)	189 (91.75%)	362 (91.88%)	*p* = 1
	Yes	15 (7.98%)	17 (8.25%)	32 (8.12%)	
I never use social media	No	187 (99.47%)	200 (97.09%)	387 (98.22%)	*p* = 0.124
	Yes	1 (0.53%)	6 (2.91%)	7 (1.78%)	
ORTO15	No orthorexic behaviors	114 (60.64%)	149 (72.33%)	263 (66.75%)	p = 0.019 ^*^
	Risk of orthorexic behaviors	74 (39.36%)	57 (27.67%)	131 (33.25%)	
EAT26	Low	171 (90.96%)	186 (90.29%)	357 (90.61%)	*p* = 0.957
	High	17 (9.04%)	20 (9.71%)	37 (9.39%)	

*N* number of patients, *SD* standard deviation, *p* level of significant, *BMI* Body Mass Index, *EAT* 26 Eating Attitudes Test, *PrEP* Pre-Exposure Prophylaxis, p—Mann–Whitney test for quantitative variables, chi-squared or Fisher's exact test for qualitative variables

^*^ statistical significant (*p* < 0.05)

Approximately 80% of participants worked full-time, 60% had achieved higher education (master), 75% declared that their relationship status was single. Additionally, 80% of participants declared that they are healthy people without chronic diseases. There were statistical significant differences in age and body mass index (BMI), with Polish respondants being younger (x̅ 32.5 vs x̅ 43.1) and having a lower BMI (x̅ 24.55 vs x̅ 27.53) than the Spanish men. Significant statistical differences were also observed in terms of place of residence and level of education and employment status. Spanish men had a statistical significant increase in likelihood of being single (82% vs 65% *p* < 0.001), were omnivore, and declared that they were healthy. No significant differences were found between the use of PrEP in the two groups. The ON risk was statistically significantly higher in the Polish group (40% vs 28% *p* = 0.019). Polish gay men used the Grindr dating application more often (65% vs 35%, *p* = 0.005) than Spanish.

Evaluation of the influence of variables on risk of orthorexia are shown in Table 2. Logistic regression models (individual for each of the analysed features) indicated that significant predictors of ON occurrence are the following: age (OR = 0.964), BMI (OR = 0.895), and receiving a disability pension or being unemployed (OR = 2.965). Also, staying in an informal relationship is associated with increased risk of ON occurrence by 2.14 times compared to being single (OR = 2.138), and occasional use of PrEP is associated with increased risk of ON by more than 4.5 times (OR = 4.667). In the case of Social-Media, the use of Instagram is associated with lowers the risk of ON by 52.1% (OR = 0.479), while the use of the Grindr application increases this risk by more than 5 times (OR = 5.312).

**Table 2:** Evaluation of the influence of variables on risk of orthorexia—regression proportional hazards single model

Trait		OR	95%CI		p
Age [years]		0.964	0.944	0.984	< 0.001 ^*^
BMI [kg/m^2^]		0.895	0.848	0.944	< 0.001 ^*^
Residence	Village	1	ref		
	City up to 100 th. inhab	0.669	0.203	2.206	0.509
	City 100–500 th. inhab	1.207	0.425	3.426	0.724
	City over 500 th. inhab	0.893	0.344	2.317	0.817
Education	Primary	1	ref		
	Secondary	1.179	0.364	3.814	0.784
	Bachelor's degree	0.87	0.281	2.693	0.809
	Master	0.55	0.188	1.609	0.275
	Doctorate	2.571	0.64	10.338	0.183
Employment status	Full-time paid job	1	ref		
	Part-time paid job	2.223	0.629	7.864	0.215
	Own business	1.245	0.619	2.502	0.538
	Pension, unemployed	2.965	1.001	8.783	0.05 ^*^
	Student	1.235	0.549	2.778	0.609
Relationship status	Single	1	ref		
	In a partnership	2.138	1.225	3.732	0.007 ^*^
	Divorced	0.507	0.107	2.394	0.391
	Married to a man	1.073	0.447	2.579	0.874
	Married to a woman	2.281	0.316	16.45	0.413
Nutrition model	Omnivore	1	ref		
	Vegan, vegetarian	3.077	1.279	7.406	0.012 ^*^
	Other	0.71	0.189	2.672	0.613
Self reporterd Health assessment	I am healthy, I do not use healthcare	1	ref		
	I am ill with chronic diseases, I use healthcare	0.757	0.375	1.53	0.438
	I have health problems but I do not receive health care	2.019	0.737	5.53	0.172
	I have mental health problems, I use psychological or psychiatric help	1.101	0.396	3.06	0.853
PrEP use	Never	1	ref		
	Always, once a day	0.25	0.057	1.105	0.067
	Occasionally	4.667	1.186	18.362	0.028 ^*^
Facebook use		0.916	0.563	1.49	0.724
Instagram use		0.479	0.279	0.822	0.008 ^*^
Twitter use		2.001	1.161	3.446	0.012 ^*^
Grindr use		5.312	3.373	8.365	< 0.001 ^*^
Other social media use		0.905	0.416	1.972	0.802
EAT26	Low	1	ref		
	High	1.097	0.539	2.232	0.798

*OR* odds ratio, *CI* Confidence interval, number of patients, *p* level of significant, *BMI* Body Mass Index, *EAT 26* Eating Attitudes Test, *PrEP* Pre-Exposure Prophylaxis

^*^statistical significant (*p* < 0.05)

The multivariate analysis showed that Grindr usages (OR: 4.72; *p* < 0.001) correlated with higher risk of ON. Higher BMI (OR = 0.913) and daily use of PrEP (OR = 0.142) is associated with lower risk of ON (see Table 3).

**Table 3:** Evaluation of the influence of variables on risk of orthorexia—regression proportional hazards multi-factor model

Trait		OR	95%CI		p
Age	[years]	0.993	0.967	1.021	0.638
BMI	[kg/m^2^]	0.913	0.851	0.98	0.011 ^*^
Nutrition model	Omnivore	1	ref		
	Vegan, vegetarian	2.081	0.739	5.857	0.165
	Other	0.548	0.128	2.352	0.419
PrEP use	Never	1	ref		
	Always, once a day	0.142	0.03	0.674	0.014 ^*^
	Occasionally	2.414	0.529	11.016	0.255
Instagram use		0.596	0.317	1.12	0.108
Twitter use		1.751	0.917	3.343	0.09
Grindr use		4.723	2.89	7.72	< 0.001 ^*^
Professional activity		0.629	0.175	2.257	0.477
Being in relationship		1.218	0.684	2.169	0.503

*OR* odds ratio, *CI* Confidence interval, number of patients, *p* level of significant, *BMI* Body Mass Index, *PrEP* Pre-Exposure Prophylaxis

^*^statistical significant (p < 0.05)

## Discussion

The first aim of the present exploratory study was to identifying demographic factors associated with ON behaviour among a sample group of Spanish and Polish identifying gay men. Approximately one-third (33%) of respondents showed risk of orthorexic behaviors. Of those who showed risk for ON, 56% were Polish, which tracks with previous literature which has shown higher rates of ON-related behaviors and practices among citizens of Western and European countries, where cultural concepts of health are more pervasive and potentially harmful in their influences on personal behaviors [25]. ON incidence in the general population is estimated to range from 6.9% to 57.6% [26, 27]. The presence of symptoms in the Polish group (40%) was statistically significantly more frequent that in the Spanish group (28%) (Chi2 *p* = 0.031).

Specifically, dietary patterns that are considered more “westernized” in nature are associated with greater risk and prevalence of ON-related behaviors [28], particularly those dietary patterns that are often associated as “healthier” in nature. Gay men in general have shown to have higher rates of sub-clinical and full-syndrome eating disorders and body dysmorphia than heterosexual adults, with overall rates of ON-related risks being higher in the LGBT population [29, 30]. The impact of cultural differences on ON is being debated in literature [31]. Furthermore, the criteria for the recent ON definition have already taken into account cultural aspects [32].

The second aim of this study was to identify predictors of orthorexia nervosa that may be unique to gay men. The most important predictors of orthorexia nervosa found (significant in both univariate and multivariate analyses) were: low BMI and the use of Grindr. Lower BMI being associated with higher risks for ON is consistent with findings preivously reported in the literature [33, 34]. This may be due to the relationships between a lower BMI and following restrictive dietary behaviours, which may be linked with beliefs and behaviours that idealize “thinness” in the pursuit of a supposedly healthier lifestyle [35].

Grindr usage was also found to negatively influence user body image and satisfaction through its impact on personal feelings of sexual objectification, weight stigma, and social comparisons [36]. In this current study, Polish men were found both have higher risk of ON-related behaviours, and to use Grindr more often than Spanish men. Part of this relationship may be influenced by increases stressors related to hypersexualized masculinity and the need to present a specific body type to increase likelihood of sexual connection when suing Grindr [37]. In particular, the visual/pictorial grid that is used by the Grindr app when displaying profiles may particularly contribute to this need for a specific form of physicality. The pursuit of a hypermasculine body, in particular muscularity, can lead to disordered eating behaviours, where the goal is to have a higher muscle mass percentage and a lower total body fat percentage [38]. This can lead to a concerning set of pressures regarding the potential development of disordered eating habits and behaviours, such as those found in ON. It is also important to note that the approval seeking that may be at play regarding Grindr usage may also explain the possible association between being in a partnership and ON risk, as in both cases the desire to be sexually and physically attractive and desirable may increase the likelihood of engaging in disordered eating behaviours.

Of note is the effect of PrEP, as daily use is associated with lower risk of ON, and occasional use increases the risk of ON. Previous research has shown that daily use of PrEP is associated with reduced sexual anxiety and improved levels of satisfaction and esteem, which may have a positive influence on body image satisfaction and reduced risk for ON [39]. Those men who participate in regular HIV-prevention strategies such as a frequency for HIV testing have been found to be engage in greater use of PrEP, and men who self-report a healthier level of body image satisfaction may be more inclined to speak with their partners about HIV-testing habits [40–42]. However, it is important to note that this reduction in the risk for ON is only true in multivariate analyses, and that the influence of occasional use on increased risk for ON was only found in univariate analyses. The role of PrEP, therefore, requires more careful study. Other predictors that are significant in univariate analyses (age, unemployment, etc.) do not seem to have a direct impact on ON. Results using the ORTO-15 show that Polish gay man had statistically significant higher risk of ON. This particular finding may suggest cross-cultural differences in ON attitudes and behaviours. However, it should be noted that the possible influence of cultural differences is currently not well understood, especially considering the lack of data about this issue at this time. One possible reason is likely due to differences in cultural concepts that concern dietary habits and overall health beliefs and attitudes, but to truly understand the possible impact of cultural differences requires further investigation.

## Study limitations

There are specific study limitations that are important to highlight. The sample size, although sufficient to assess the main objectives of the study, could have been improved by the addition of more participants. In some parameters the group size was too small to draw accurate conclusions. These analyses are cross-sectional, so directionality of these relationships cannot be inferred, so no causal inferences may be made in this cross-sectional survey research. Another limitation is that ORTO-15 is tool to assess severity of ON symptoms. It's difficult to define unequivocally whether a person with a high score in the questionnaire represents only a specific life-style characterizing in a healthy eating habits or this person has a medical problem resulting from pathological eating patterns. An additional limitation is that the study was conducted only via an online platform, possibly leading to the exclusion of some groups of potential participants (e.g., people without the internet or the elderly who usually use the internet rarely). Lastly, researchers did not have data from study participants related to auto-immune diagnosis, gastrointestinal problems, health-related anxiety, and other impacting psychological constructs that can be related to ON behavior. Future research in study ON in populations would benefit from this additional study and analysis.

## Conclusions

The most important predictors of orthorexia nervosa in gay man are low BMI and the use of Grindr. The effect of daily usage of PrEP is associated with lower risk, and occasional use is associated with increased risk, of orthorexia nervosa. Healthcare professionals and public health researchers working with gay men should be aware of the unique ways in which ON may present itself in the gay male population. Further research is needed to better understand the potential links between Grindr usage and lifestyle behaviours such as PrEP usage and dietary habits, in particular the potential harmful effects on body image satisfaction that can lead to ON-related behaviours.

## Supplementary Information

**Additional file 1.**

## Data Availability

The datasets used and/or analysed during the current study available from the corresponding author or can be consulted at the link in the supplementary document 1 (Additional file 1).

## Abbreviations

ON: Orthorexia Nervosa
PrEP: Pre-exposure prophylaxis
EAT-26: Eating Attitudes Test 26
OR: Odds Ratio
NCD's: Chronic Non-Communicable Diseases
ED: Eating Disorder
DSM: The Diagnostic and Statistical Manual of Mental Disorders, Fifth Edition
LGBT: Lesbian, gay, bisexual, transgender
ORTO-15: Orthorexia Nervosa ORTO-15
CEImLAR: Committee on Ethics and Research medicinal of La Rioja
STROBE: Strengthening the Reporting of Observational Studies in Epidemiology
EPV: Events Per Variable
SD: Standard Deviation
BMI: Body Mass Index
