# Supplementary material for: Orthorexia nervosa in gay men—the result of a spanish-polish eating disorders study
Source: BMC Public Health. 2023 Jan 9;23:58. doi: 10.1186/s12889-022-14943-7 (PMC9830745; doi:10.1186/s12889-022-14943-7)
Supplement: Supplementary file 1 — Additional file 1. [file 12889_2022_14943_MOESM1_ESM.docx]

**Availability of data and materials**

The datasets used and/or analysed during the current study available from the corresponding author or can be consulted at the link:

<https://1drv.ms/x/s!AiviG_zdAuoDnAydFYXGZlstjX6C?e=YGd5Hw>
